# 

**Published:** 1842-04

**Authors:** 


### CONTENTS

OF NO. IV.

---

### ORIGINAL COMMUNICATIONS.

### ESSAYS AND CASES.

Art. I.—A Clinical Lecture on the Nature, Causes, and Treatment of Strabismus; delivered at the Louisville Marine Hospital, February, 1842. By S. D. Gross, M. D., Professor of Surgery in the Louisville Medical Institute. 241

Art. II.—Phlegmasia Dolens. By John Hardin, M. D., of Horse-Well, Kentucky. 265

### BIBLIOGRAPHICAL NOTICES.

Art. III.—Proceedings of the Physiological Temperance Society of the Medical Institute of Louisville. 1842, pp. 16. 267

Art. IV.—Twenty-fourth Report of the McLean Asylum for the Insane. By Luther V. Bell, M. D., Superintendent. Boston, 1842, p. 40. 271

Art. V.—The Principles and Practice of Obstetric Medicine and Surgery, in reference to the Process of Parturition: Illustrated by 142 figures. By Francis H. Ramsbotham, M. D., Consulting Physician in Obstetric Cases to, and Lecturer on Obstetric and Forensic Medicine at, the London Hospital, Physician to the Royal Maternity Charity, Obstetric Physician to the Eastern and Tower Hamlets' Dispensaries. First American Edition, revised: pp. 458, large octavo. Lea & Blanchard, Philadelphia, 1842. 278

Art. VI.—The Practice of Medicine; or a Treatise on Special Pathology and Therapeutics. By Robley Dunglison, M. D,, Professor of the Institutes of Medicine, &c., in Jefferson Medical College, Philadelphia; Lecturer on Clinical Medicine, and attending Physician at the Philadelphia Hospital, &c., &c. In two volumes, pp. 572—750. Philadelphia: Lea & Blanchard, 1842. 282

SELECTIONS FROM AMERICAN AND FOREIGN JOURNALS.

Lugol's Lectures on Scrofula. - - - - - 285
Extract from a Paper on the Treatment of Varicose Veins by the needle and twisted suture. - - - 288
Quackery and Humbug.—Inflammation. - - 292
Detection of Arsenic Acid. By M. Elsner. 293
Pulse of Children at the Breast. - - - 294
Fibrin, Albumen, Casein, as Elements of Nutrition. - 294
On Milk. - - - - - - 296
A New Medical Journal in Boston. - - - 297
Six Cases of Diarrhœa treated with Monesia. - - 297
Kiestein as an Evidence of Pregnancy. - - - 299
M. Dubois on the Auscultatory Signs of Pregnancy. - 301
Division of the Muscles of the Eye in certain cases of Blindness. 302
State of the Urine during Pregnancy and Disease. - 304
Lapis Divinus in Otorrhœa. - - - - 305
On Dislocation of the Crystalline Lens. - - 307
Memoir of a Gentleman born blind and successfully operated on in the 18th year of his age. By Dr. Franz. - 308
On the Injuries to Health occasioned by breathing Impure Air in close apartments. - - - - 310
Cases of Dislocation of the Clavicle backwards. - - 314

ORIGINAL INTELLIGENCE.

Editorial Notice. - - - - - 317
Medical Convention of Ohio. - - - - 317
School for the Blind in Louisville.—Petty Misrepresentations. 317
Disease of the Tongue and Mouth. - - - 319

